# Experimental investigations on graphene oxide/rubber composite thermal conductivity

**DOI:** 10.1038/s41598-020-72633-z

**Published:** 2020-09-23

**Authors:** Joanna Wilk, Robert Smusz, Ryszard Filip, Grzegorz Chmiel, Tomasz Bednarczyk

**Affiliations:** 1 Department of Thermodynamics, Rzeszów University of Technology, Al. Powstańców Warszawy 12, 35-959 Rzeszow, Poland; 2 Department of Materials Science, Rzeszów University of Technology, Al. Powstańców Warszawy 12, 35-959 Rzeszow, Poland; 3 GUMET Company, ul. Kolejowa 12, 23-200 Kraśnik, Poland

**Keywords:** Engineering, Materials science

## Abstract

Graphene oxide/rubber composites were experimentally investigated for obtaining their thermal properties. Three kinds of the composite matrix material have been used: NBR, HNBR and FKM. The reduced graphene oxide in the form of crumped flakes has been applied as the filler influencing on thermal conductivity of the composites. Two values of graphene oxide weight concentration have been taken into account in the investigation. Thermal conductivity of the composites and basic matrix has been measured by the professional apparatus with the use of the guarded heat plate method. Before measurements the preliminary tests using the simplified comparative method have been performed. The results obtained, both from preliminary tests and using the guarded heat plate method, show an increase in thermal conductivity with increasing the reduced graphene oxide content in the composite. The experimental investigation allowed to determine not only the increase in thermal properties of graphene oxide/rubber composites compared to the basic matrix, but also the absolute values of thermal conductivities. Additionally, the SEM analysis showed that the tested composite samples contain agglomerates of the rGO nanoparticles. The occurrence of agglomerates could affect the composite thermal properties. This was noticed in the comparatively measurements of the temperature of different composites during the heating of samples tested. The maximum enhancement of thermal conductivity obtained was about 11% compared to the basis matrix of the composites tested.

## Introduction

The rubber is used extensively in many engineering applications. To improve the material properties rubber-based composites are formed. Both the natural rubber and the synthetic rubber copolymers are used as a basic matrix in composite materials. One of the rubber applications is the use as a material of the roller bearing seals. Nanoplatelets derived from different forms of carbon have recently attracted attention as an useful filler in rubber composites. The filling of the composite with nanoplatelets improves the mechanical properties of the roller bearing seals. Additionally thermal properties are also enhancement which contributes more intensive heat dissipation generated in the bearing. The different form of carbon are used as the filler in rubber-based composites. There are: graphene oxide^1–7^, graphene and graphite^8–12^ or carbon black^13^. Thermal properties of the composites considered have been studied among others in^2–4,7^ where the authors reported the results of thermal conductivity investigations for the graphene oxide/rubber composites. The authors in^2^ present the results of measurements of thermal conductivity of bromobutyl rubber composites with the ionic liquid modified graphene oxide. Weight concentrations of the graphene oxide in the composites tested were: 1; 2; 3 and 4%. The authors reported a maximum one, threefold improvement in thermal conductivity with 4% content of graphene oxide, compared to the basic matrix. The dependence of the composites thermal conductivity on graphene oxide concentration was approximately linear. In turn the study^3^ includes the results of thermal conductivity measurements in the case of graphene oxide/rubber composite with the basic matrix of carboxylated acrylonitrile butadiene rubber. The authors investigated the graphene oxides with different oxidation degrees. The graphene oxide content in the composites considered was 4 phr (parts per hundred rubber). The authors reported the increase in thermal conductivity of the composite in the range from 10.1 to 27.2% depending on the graphene oxide kind. The article^4^ deals to the graphene oxide/natural rubber composite which was prepared by a novel in-situ method. The authors reported 36% increasing in thermal conductivity of the composite compared to the pure rubber. Despite the low content of the reduced graphene oxide (0.1 phr) the high enhancement of thermal properties has been observed. In turn the work^7^ deals to investigations of the interfacial thermal transport in graphene oxide/butadiene-styrene-vinyl pyridine rubber composites by molecular dynamics simulation. The authors concluded on the best oxidation degree of graphene oxide about 15%. For this value the highest thermal conductivity of the composites is achieved.

The another above quoted articles^8–13^ included investigations in thermal conductivity of graphene/rubber composites or graphite and carbon black as fillers. In all cases considered the increase of thermal conductivity has been observed.

The main goal of the present work was to determine the thermal conductivity of the selected reduced graphene oxide/rubber composites applied as a material on roller bearing seals. Because of practical applications of the materials considered the knowledge of their thermal properties is important and useful. Enhancement in thermal conductivity by adding the filler in the form of the reduce graphene oxide improves the heat rejection from the bearings. Matrix materials of tested composites were acrylonitrile butadiene rubber (NBR), hydrogenated acrylonitrile butadiene rubber (HNBR) and fluoroelastomer (FKM). Due to the heterogeneity of materials investigated, special attention was paid to the measurement technique. Thermal conductivity of the composites and the basic matrix has been tested with the use of the guarded heat plate method. Discussion on the measurements techniques for thermal conductivity of not homogeneous materials confirmed the validity of the method applied.

## Experimentation

### Materials characteristics

**Figure 1:**
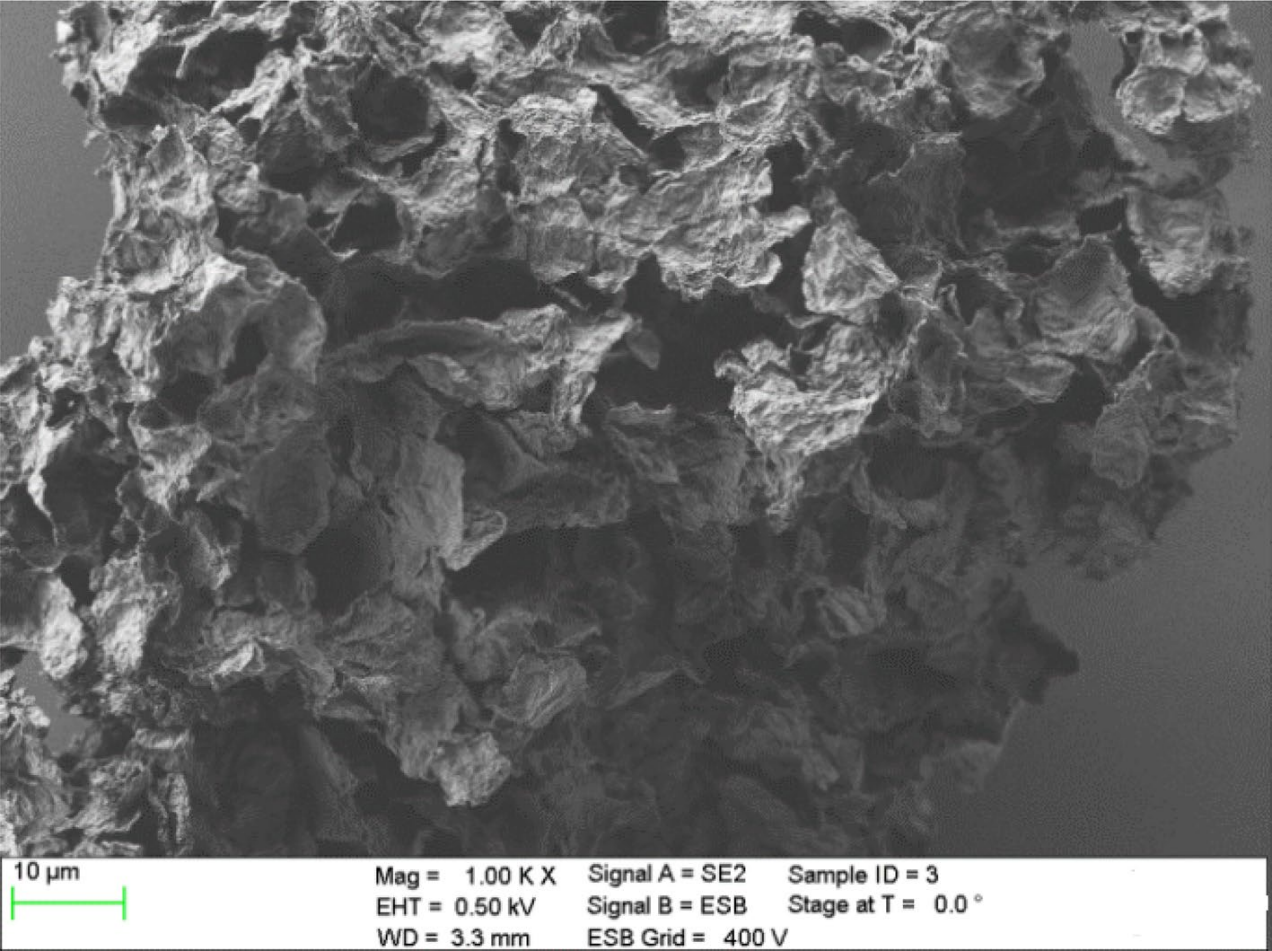
SEM image of the reduced GO applied.

The reduced graphene oxide (rGO) used as the filler influencing on thermal conductivity of the composites investigated has been purchased from Institute of Electronic Materials Technology, Warsaw, Poland. The considered graphene oxide has been reduced with sodium hypophosphite. The basic properties of the rGO included: the appearance as the grey to black powder, hydrophobic and stable in air, the bulk density equal to 0.019 g/cm^3^, the specific surface area of 266 m^2^/g, the specific electrical conductivity − 24 S/cm^2^. The scanning electron microscope (SEM) images of the graphene oxide nanoparticles are presented in Fig. 1.

**Figure 2:**
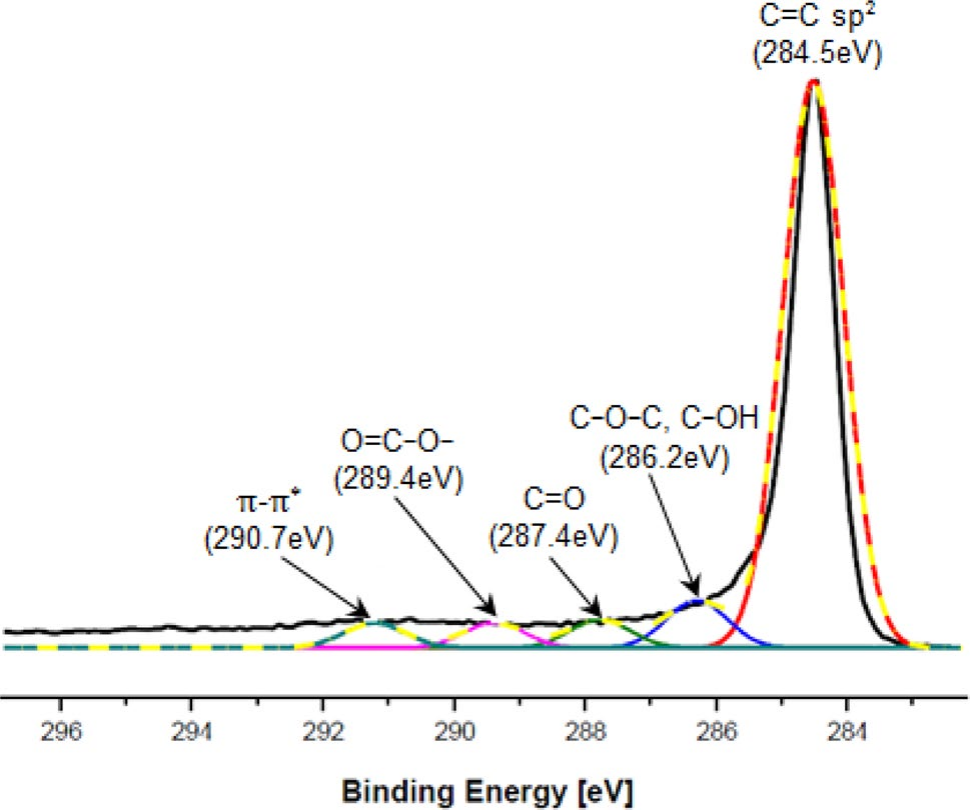
C1s XPS spectrum of the reduced GO.

As can be seen the rGO material consists of thin, crumpled flakes with diameter of a few micrometers which forming the loose, disordered, crumbling structures. The X-ray photoelectron spectroscopy (XPS) of the rGO considered is shown in Fig. 2. The C1 s spectrum of rGO shows a major peak at 284.5 eV which is attributed to the sp^2^ carbon of C=C bonding^11^. Other peaks are much smaller what indicates the considerable deoxygenation by the reduction process of the graphene oxide^1^. The XPS spectra obtained is comparable with the another types of rGO also applied as thermal conductive filler in rubber graphene composites^1,4^.

The reduced graphene oxide applied as the filler in the rubber composites has a high thermal conductivity. The literature data gives values of GO thermal conductivities from several to over two thousands W/(mK)^14,15^ depending on the form of GO. In the case of the rGO used in the investigations the value of thermal conductivity has been estimated on the basis of the model of the effective thermal conductivity equation for two-component material. The results obtained have been included in Chapter 3—Results and discussion.

As the basic matrix of composites three types of rubber have been used. There were: acrylonitrile butadiene rubber (NBR), hydrogenated acrylonitrile butadiene rubber (HNBR) and fluoroelastomer (FKM). For the fabrication of rGO/rubber composites the conventional mechanical method enabling the relatively uniform dispersion of the rGO flakes in the rubber matrix has been used. The suitable amount of rGO in the form of crumpled flakes were directly dispersed to the basis rubber during the rolling process with the use of two-roll mill calendaring. The process has been performed on the laboratory roll-mill of 400 mm width and 150 mm diameter cylinder at temperature 70 °C for about 10 min. The two-roll mill applied has provided the most homogenously disperse the reduced graphene oxide in the rubber examined. After the rolling process the weight of the composite formed has been determined and the weight concentration of rGO has been obtained from the equation

1 $$w = \frac{{m_{rGO} }}{{m_{BM} + m_{rGO} }},$$

where *m*_*BM*_ is a mass of basic matrix.

The density of the graphene oxide/rubber composites have been determined using the volumetric weighing method. An accuracy of density measurements was estimated at ± 0.003 g/cm^3^. Before thermal investigations the experimental research on some mechanical properties of the composites considered have been performed. As the results the values of indentation hardness and elastic modulus have been received. Mechanical properties were investigated with the use of Ultra Nano Indentation Tester. The basic characteristics of the composites tested are included in Table 1. The results of the investigations showed an improvement in the properties, both of elastic modulus and indentation hardness of the rGO/NBR and rGO/HNBR rubber composites, with the increase of rGO concentration. In the case of rGO/FKM rubber composites the increase in rGO concentration did not cause the improvement of mechanical properties.

**Table 1:** Basic characteristic of rGO/rubber composites.

Symbol	Basis matrix	*w* [%]	*ρ* [g/cm^3^]	*HIT* [MPa]	*E* [GPa]
N0	NBR	0	1.273	1.216	0.010
N1	NBR	1.5	1.276	1.729	0.016
N2	NBR	2.5	1.808	1.801	0.017
H0	HNBR	0	1.199	1.574	0.025
H1	HNBR	2.5	1.207	2.156	0.032
F1	FKM	1.5	1.885	1.519	0.016
F2	FKM	2.5	1.885	1.312	0.013

**Figure 3:**
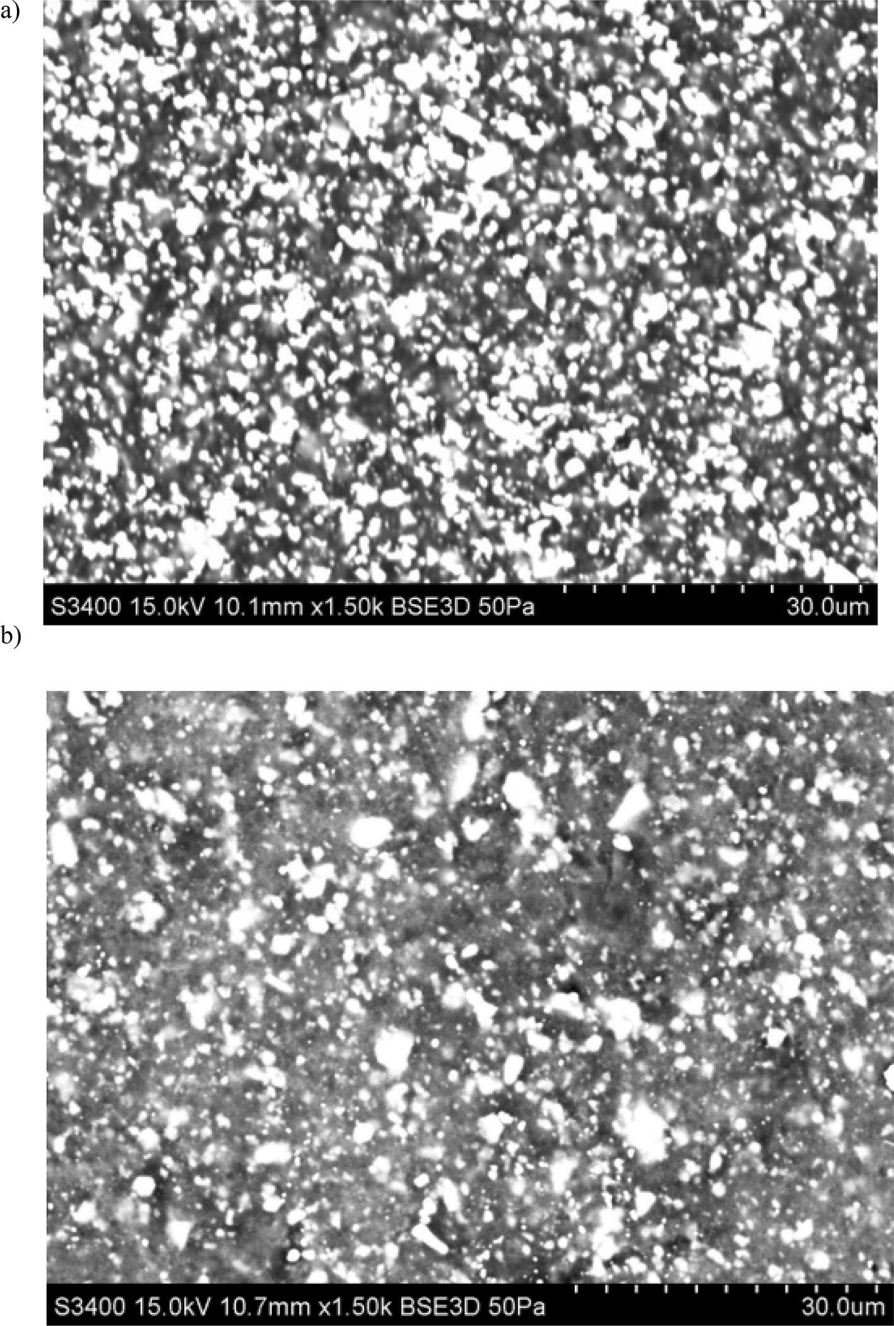
Surfaces of tested materials: (**a**) N2 rGO/rubber, (**b**) F2 rGO/rubber composite.

**Figure 4:**
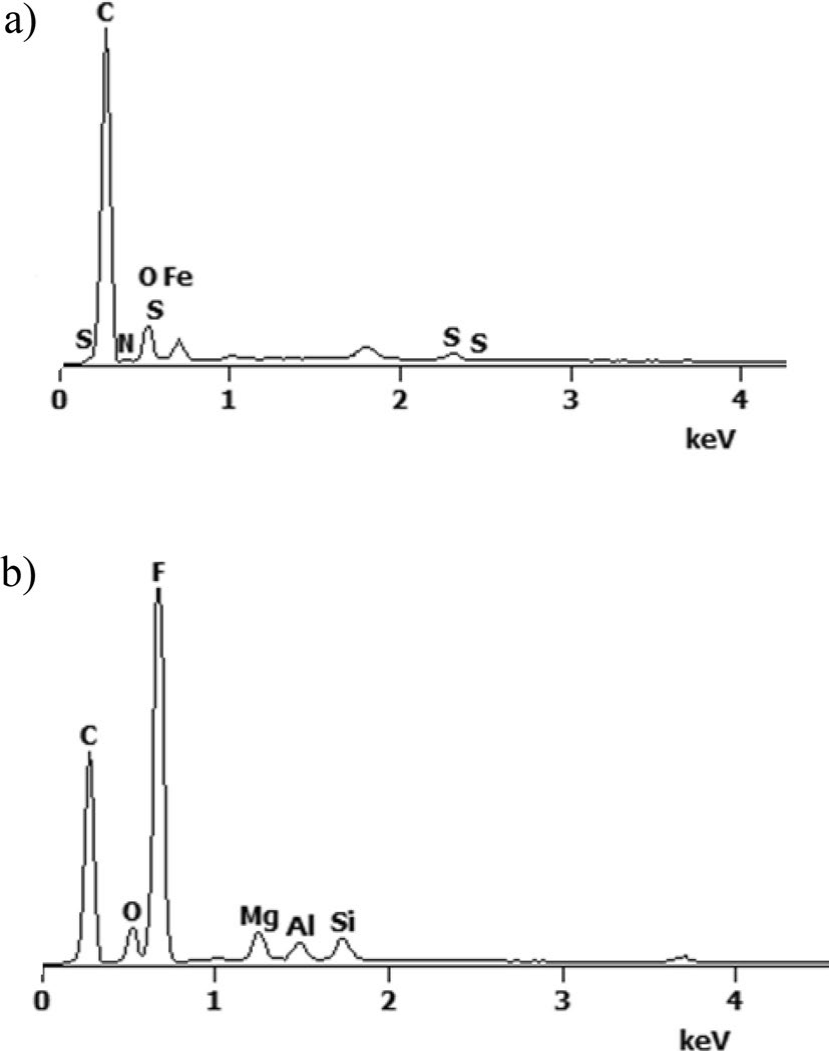
EDS spectrum of: (**a**) N2 rGO/rubber and (**b**) F2 rGO/rubber composites.

A chemical characterisation of the composites have been performed using the energy-dispersive X-ray spectroscopy (EDS). The samples for EDS analysis have been prepared directly from the rGO/rubber composites. Before investigations the external surfaces of samples tested were cleaned and degreased in ultrasonic bath of ethanol. Figure 3 presents the exemplary images of the surface tested with the selected area of the chemical composition analyse. In turn the corresponding results of the qualitative analysis of the chemical compositions of the materials investigated are presented in Fig. 4. The images in Fig. 3 clearly show the differences in the structure of the NBR and FKM composites. The composite with the acrylonitrile butadiene rubber matrix N2 has a more compact and uniform structure than the F2 composite with the fluoroelastomer matrix material. Thus, rGO/FKM composites are characterized by higher porosity than rGO/NBR ones. This affects the thermal conductivity of the materials considered. Thermal conductivity of of N2 rGO/rubber composite is more than 50% higher than the conductivity of F2 what has been presented and discussed in the next part of the article (see Table 3).

As can be seen in Fig. 4 the peak attributed to oxygen indicates of a pronounced content of reduced graphene oxide in the basis matrix. The other elements as S, Mg, Al, Si, Fe are the components of additives using during the basis matrix forming process. Figure 4b also shows that the peak attributed to fluorine has a major value, what is a main difference between NBR and FKM materials used in investigations.

**Figure 5:**
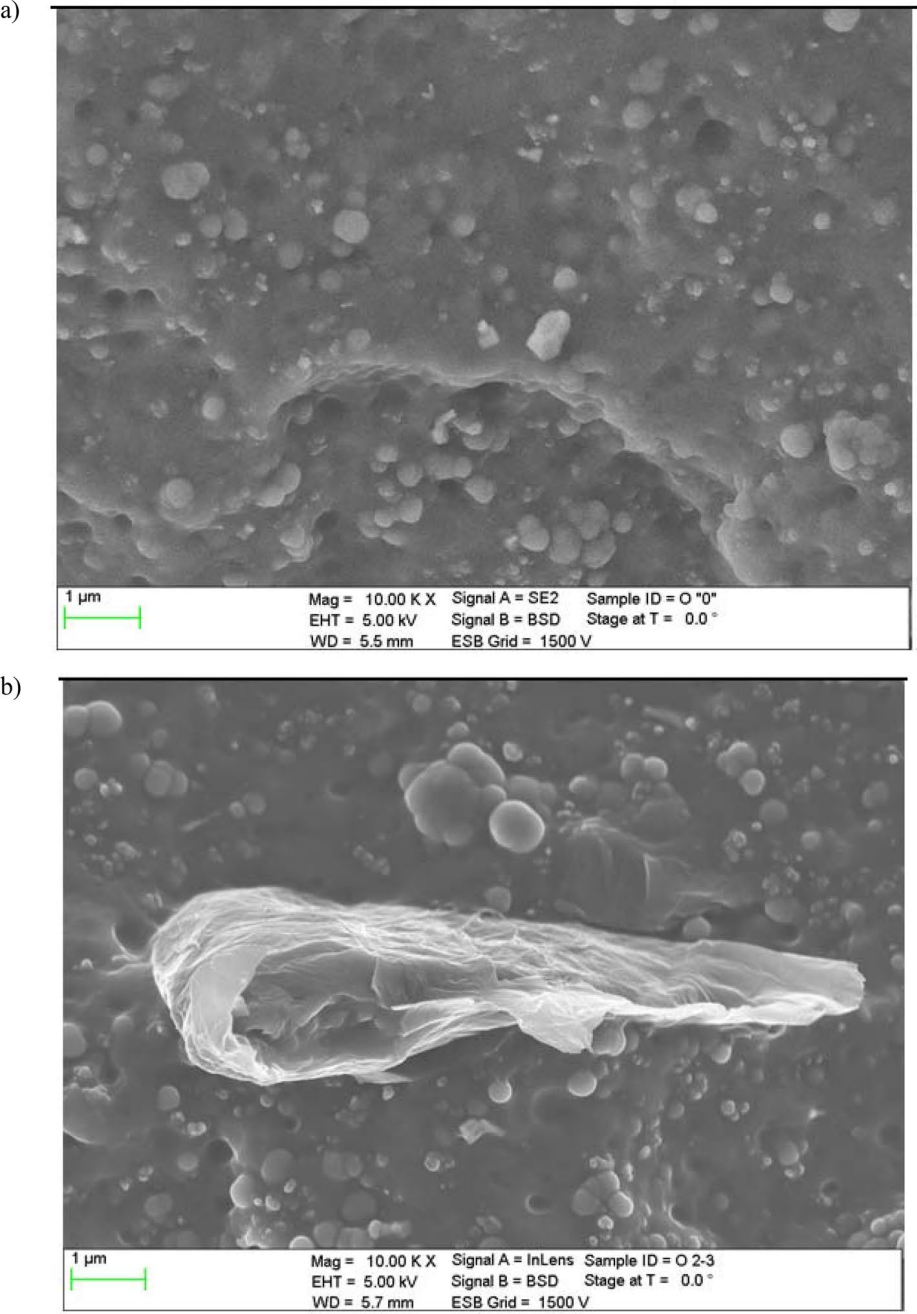
SEM images of cross-sectional area: (**a**) NBR-N0 rubber, (**b**) N2 rGO/rubber composite.

**Figure 6:**
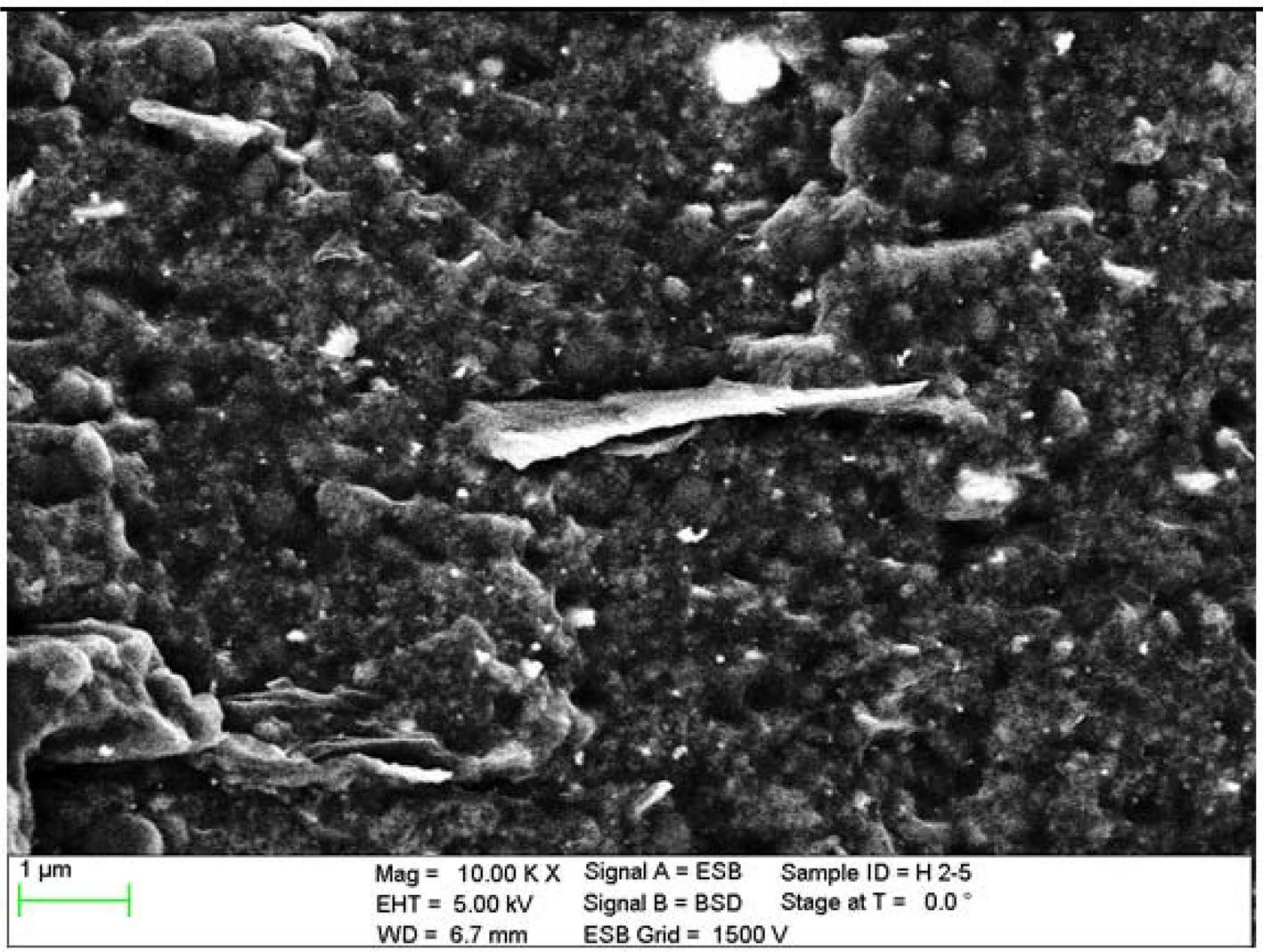
SEM image of cross-sectional area of H1 rGO/rubber composite.

**Figure 7:**
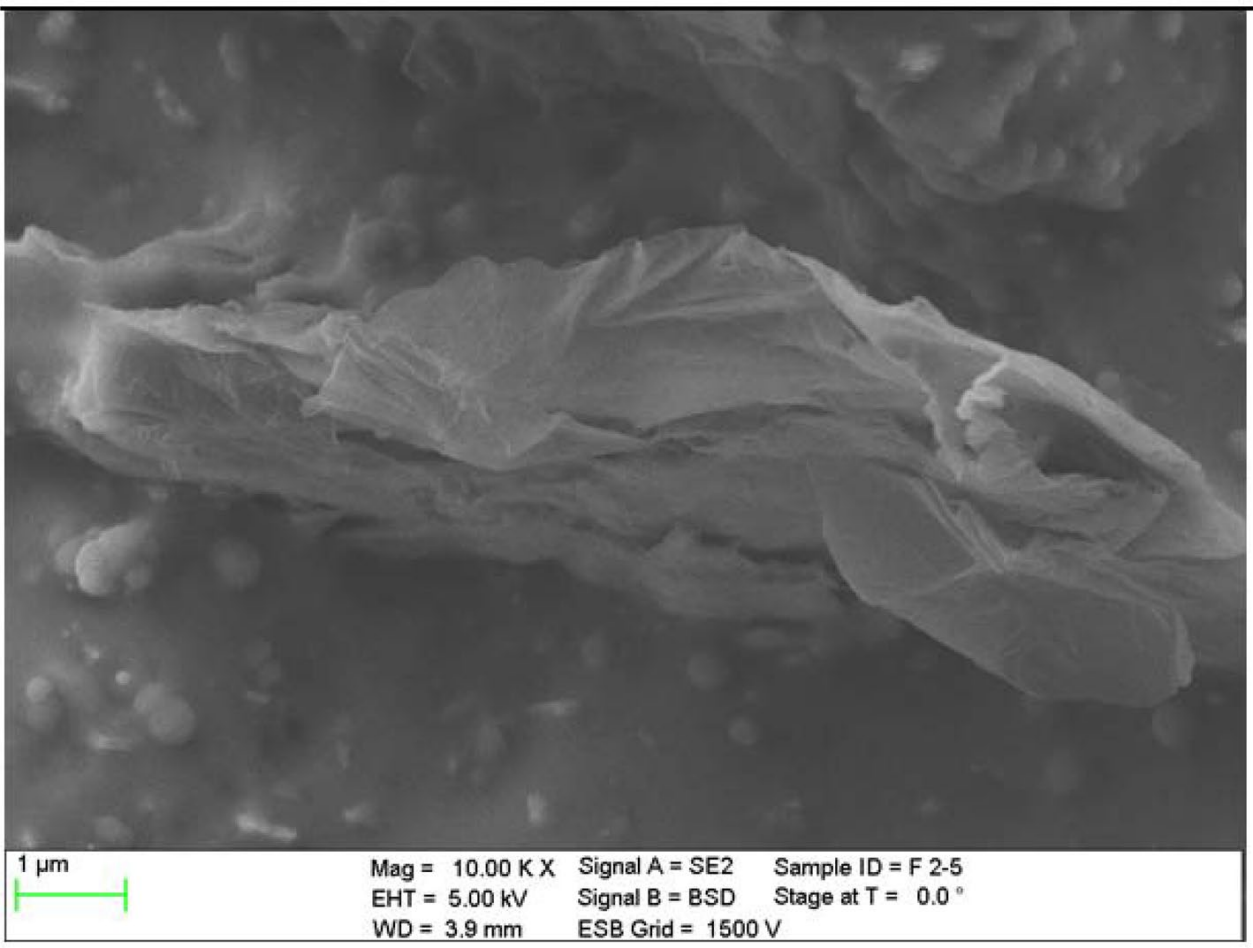
SEM image of F2 rGO/rubber composite cross-sectional area.

The next test enabling the characterization of rGO/rubber composites was the scanning electron microscope analysis of the cross section of the materials tested. Before investigations the samples were cleaned and degreased in ultrasonic bath of ethanol. Figures 5, 6 and 7 present exemplary SEM images of cross-sectional areas of the basis matrix and rGO/rubber composites. In the case of the composites the images of the material samples with the same rGO concentrations have been presented.

Scanning electron microscope observations of the filler dispersion and fracture surfaces have indicated that in the case of NBR and HNBR composites the relatively uniform distribution of reduced graphene oxide flakes occurs. However, there are agglomerates and their clusters. In the case of HNBR matrix material, where the hydrogenation process increases temperature resistance, the rGO flakes are less visible than for NBR. This makes it difficult to analyze the distribution of nanoflakes.

In turn, in rGO/FKM rubber composites, the rGO flakes are even less visible, especially at low magnifications. The compaction of the flakes at the same rGO concentration is much smaller than in the case of NBR composites. This may indicate the presence of a large number of agglomerates of significant size.

### Thermal conductivity measurements

Measurements of thermal conductivity of the rGO/rubber composites with different graphene oxide contents were carried out in two stages. In the first stage, as the preliminary investigation, the comparative method has been performed. The obtained results encouraged further research. The second stage concerned the main measurements using the professional procedure.

**Figure 8:**
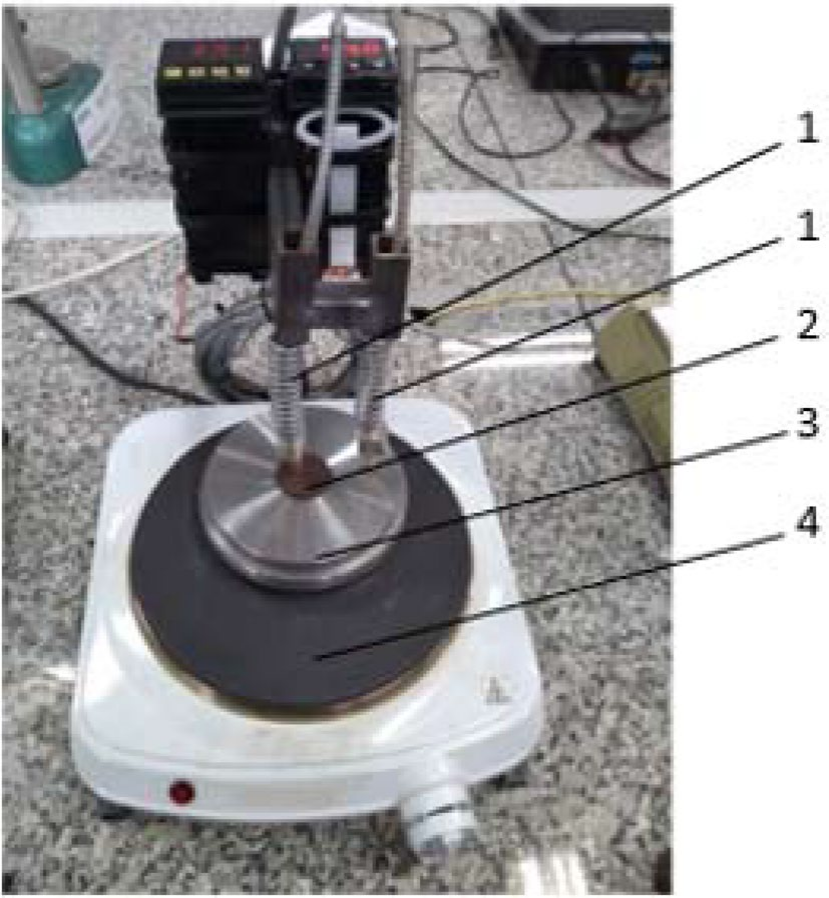
Measurement stand for the preliminary tests of thermal properties of the rGO/rubber composites: 1—resistance temperature sensors, 2—test sample, 3—steel housing of the sample, 4—electrical heater.

The preliminary investigations of thermal properties of the rGO/rubber composites had a qualitative character. They were performed on the test stand which has been constructed especially for this purpose. A view of the stand applied is shown in Fig. 8. The stand was consisted in basic elements: a hot plate, the intermediate steel element with the cavity for the sample of the investigated material, resistance temperature detectors for measurements of rubber composite sample and steel housing temperatures.

The tested samples had a circular shape with diameter *d* = 29 mm and thickness *δ* = 13 mm. Such dimensions gave a relatively large sample volume which ensured that it can be assumed the same thermal properties in all sample volume, even if the distribution of reduced graphene oxide flakes is not uniform. Temperature detectors have been placed at the depth of 5 mm. The first stage of the experiment was to investigation the changes in time of the sample temperature during the sample heating. Measurements have been performed for the rubber being the basic matrix and for the rGO/rubber composites with different graphene oxide content. The comparative analysis showed the filler influence on thermal conductivity of the composites.

The second stage of the experiment was to determining the thermal conductivity of samples tested with the use of standard method on the professional apparatus.

The literature reviewing sees that nowadays, in the measurements of thermal conductivity of rubber composites, the researches use techniques: laser flash method^10–13,16^, transient plane source method^4,8^ or transient hot wire method^17^. The alternative method applied in composite thermal conductivity investigations is a molecular dynamics simulation^7^. The laser flash method enables thermal diffusivity obtaining, therefore, for determine the thermal conductivity the specific heat and density measurements are necessary. The small size of a test sample is an additionally challenge both in diffusivity and heat capacity measurements. In the case of laser flash method the sample dimensions are about *δ* ≈ 1 mm, *d* ≈ 12 mm. In turn, measurements of the specific heat with the use of differential scanning calorimeter require samples of about 10 mg weight. In this case, if the material tested is heterogeneous, test results could be unreliable. In turn, the transient methods, both the plane source and the hot wire, require the infinite in all direction and isotropic test sample^18–20^. Ensuring these conditions is very difficult. The authors in^4^ applied the plate-shaped samples of rGO/natural rubber composite with side of 254 mm and thickness of 50 μm. Such dimensions approximate infinity in only two directions. The very small thickness of the sample could significantly affect the measurement results. The second transient method—the hot wire—requires placing the probe being the linear thermal source in the test sample. Unlike the previous method, the heat source has a relatively large dimension and in this case, if the tested material is solid, the minimizing of thermal contact resistance between the probe and the material tested is an additional difficulty. Therefore, the hot wire method is rather used for thermal conductivity measurements in liquids or bulk solids^21–26^. The another method used in thermal conductivity research is the steady state technique—guarded hot plate method (GHP)—when material sample that is analyzed is in complete thermal equilibrium. The guarded hot plate method is widely used in measurements of thermal conductivity of insulation materials, that materials characterized by high thermal resistance. Examples of recent works are^27–29^. The rGO/rubber composites, being the subject of the present work, are also characterized by high thermal resistance. Generally, the rubber—matrix of composites tested—is a poor heat conductor. It can be expected that the addition of graphene oxide will not cause very significant changes in thermal conductivity. This has been confirmed in preliminary studies. Thus it seems that the guarded hot plate method is right in rGO/rubber composites research. An additional advantage is the method accuracy. It is in the range from 3 for 8%. In the present research the authors determined the expanded uncertainty of thermal conductivity at about 6%. Comparing with the another technique—the laser flash method—an accuracy of the GHP method will be better. In the case of laser flash method the uncertainty of thermal diffusivity, specific heat and density measurements increase the uncertainty of thermal conductivity measurement.

The GHP has been used for measurements of graphene oxide/rubber composite thermal conductivity also by the other researchers^2,3^.

In the rGO/rubber composites considered in the present study, the distribution of reduced graphene oxide flakes is not uniform. Agglomerates are present in the tested material, thus, for the obtain reliable results, a relatively large material sample should be tested. Dimensions of the circular samples investigated were: *δ* = 7.65 mm for N0, N1, N2 and *δ* = 7.67 mm for H0, H1, F1, F2; *d* = 50.8 mm for all samples. It has ensured obtaining the averaged values of thermal conductivity in the entire volume of the sample. Additionally, the thermal contact resistance between test sample and the apparatus plates (interface thermal resistance) could be easily minimized. This is possible thanks to apply a compressive load on the test stack consisting of material sample and the heating and cooling plates.

**Figure 9:**
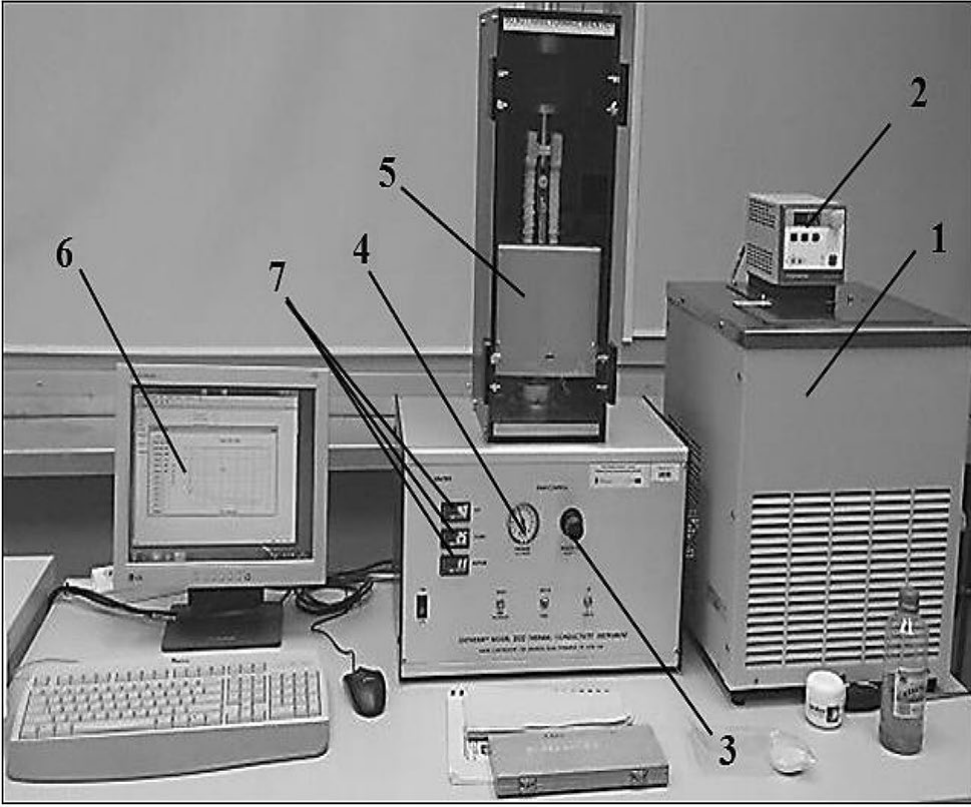
Measurement stand for thermal conductivity investigation by the guarded heat flow meter method: 1—thermostat, 2—temperature controller, 3—air pressure regulator, 4—air pressure gauge, 5—guard heater, 6—PC, 7—heater PID controllers.

**Figure 10:**
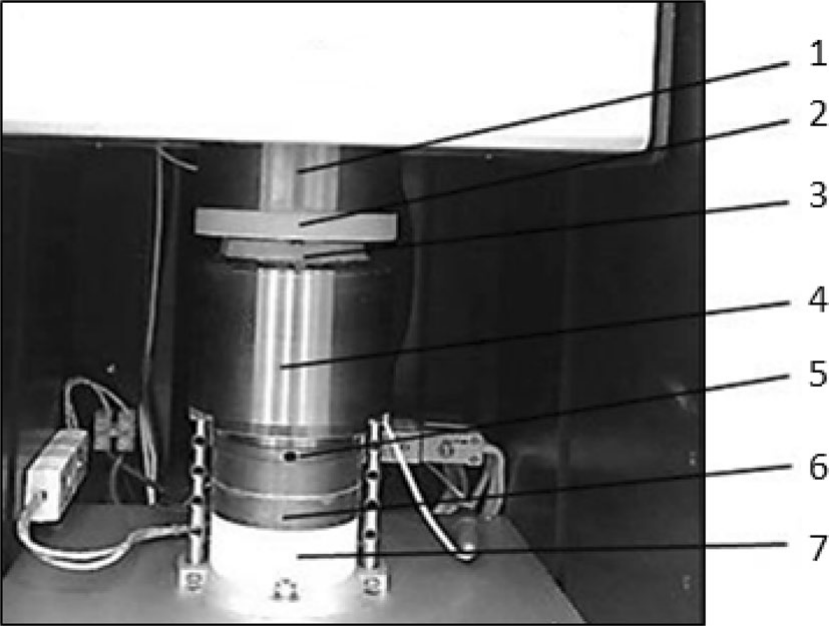
Test section of the GHP apparatus: 1—top heater, 2—upper plate, 3—test sample, 4—lower plate, 5—heat flux transducer, 6—bottom heater, 7—cooler.

Thermal conductivity of the rGO/rubber composites with different graphene oxide contents was measured by the guarded heat flow meter method with the use of the Unitherm 2022 thermal conductivity apparatus. Measurements have been performed according to the Standard^30^. The instrument applied is commonly named as a guarded hot plate apparatus (GHP). Figures 9 and 10 present views of the measurement stand and the test section with the main elements marked.

The GHP technique is widely taken as a primary method to determine the effective thermal conductivities of materials with relatively high thermal resistance. The GHP apparatus applied was consisted of a flat-plate test sample, a hot-surface assembly and a cold-surface one. The cold and hot surface assemblies have been placed above and below the test sample, respectively. The thermal interaction of the apparatus with the surroundings was prevented by the insulation material. The test sample of the composite material was held under a reproducible compressive load between two metal surfaces, each controlled at a different temperature. The lower contact surface was a part of a calibrated heat flux transducer. During the heat flow from the upper surface through the composite material to the lower surface, an axial temperature gradient was established. The measurement of the temperature difference across the sample along with the output from the heat flux transducer and the known sample thickness enabled determining thermal conductivity of the composites investigated^31^.

The thermal resistance of a composite material sample has been obtained using Fourier’s law in a steady state. Assuming thermal equilibrium the Fourier’s law applied becomes

2 $$R_{s} = \frac{{T_{u - } T_{m} }}{q} - R_{int} ,$$

where:

*R*_*s*_—thermal resistance of the composite material sample, *T*_*u*_—upper plate surface temperature, *T*_*m*_—lower plate surface temperature, *q*—heat flux through the sample, *R*_*int*_—total interface resistance between sample and surface plates.

In turn thermal resistance is defined as

3 $$R_{s} = \frac{\delta }{k},$$

where *δ* is a thickness of the sample tested.

The heat flux *q* has been determined by measuring the appropriate temperature difference across the reference calorimeter according to

4 $$q = N\left( {T_{m - } T_{L} } \right),$$

where *T*_*L*_ is the bottom heater temperature and *N* is the reference calorimeter heat transfer coefficient.

The necessary coefficient for the *q* obtaining and the value of resistance *R*_*int*_ in Eq. (2) have been determined in the calibration procedure of the GHP apparatus.

## Results and discussion

### Qualitative research

**Figure 11:**
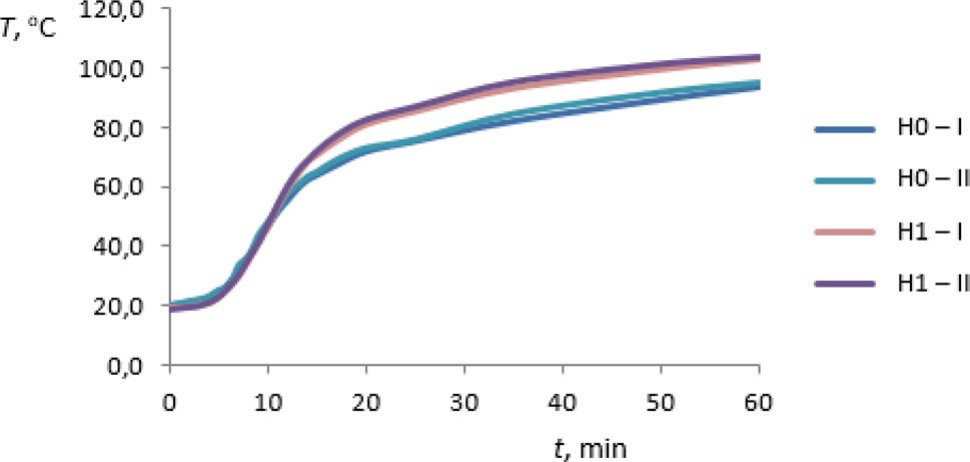
Temperature distribution of the HNBR matrix (H0) and rGO/HNBR rubber composite (H1) during the all heating time; I, II—test sample number.

**Figure 12:**
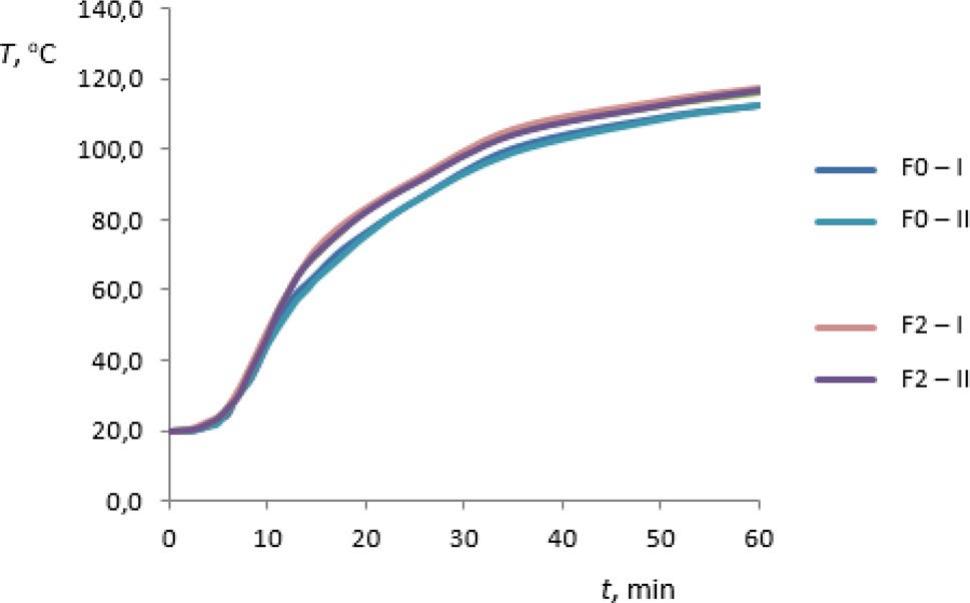
Temperature distribution of the FKM matrix (F0) and rGO/FKM rubber composite (F2) during the all heating time; I, II—test sample number.

**Figure 13:**
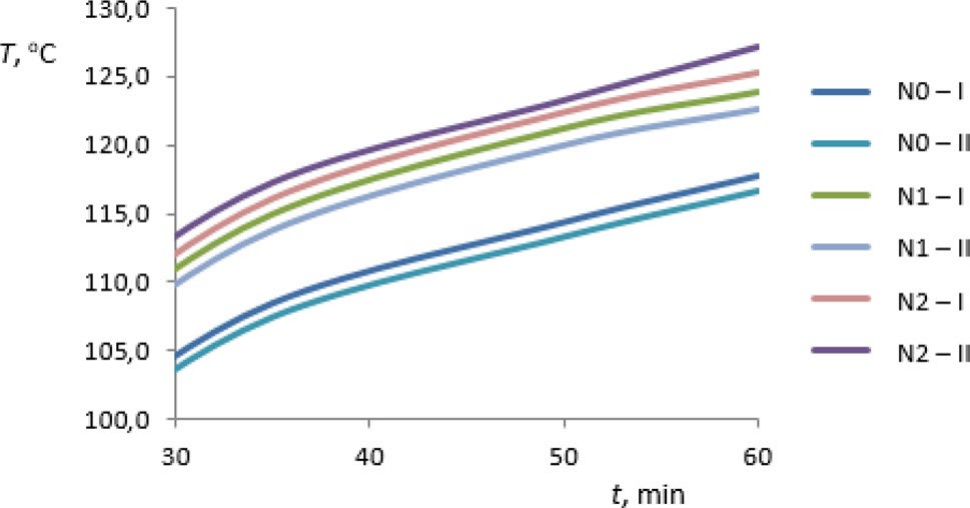
Temperature distribution of the NBR matrix (N0) and rGO/NBR rubber composites (N1, N2) during the second half of the heating time; I, II—test sample number.

**Figure 14:**
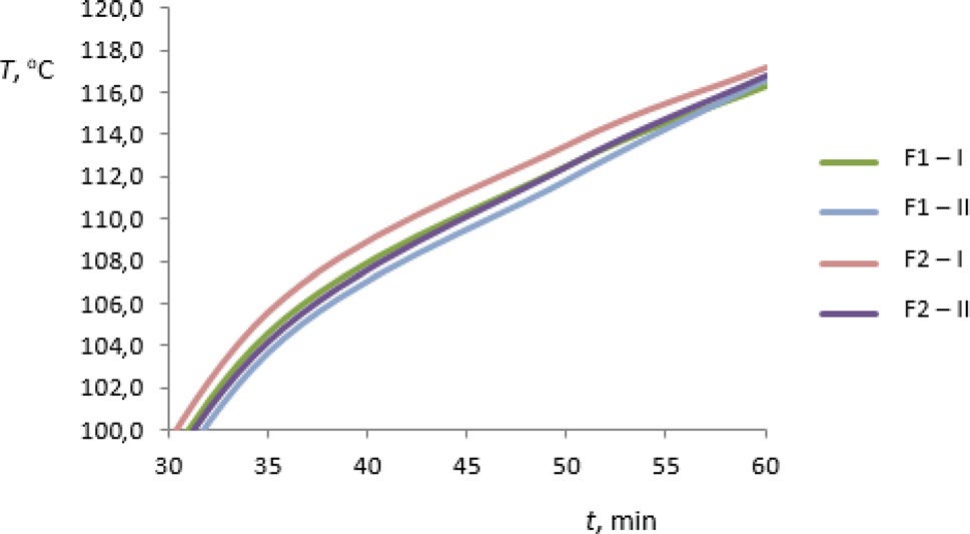
Temperature distribution of rGO/FKM rubber composites (F1, F2) during the second half of the heating time; I, II—test sample number.

The preliminary investigations of thermal conductivity of the rGO/rubber composites and of the basic matrix were carried out for the materials summarized in Table 1 and additionally for FKM rubber without graphene oxide—F0 sample. As the results the temperature distributions in sample heating time have been received. The heating process was occurring from the ambient temperature to reach the certain set heater temperature the same in all cases considered. For each case the measurements of two test samples I and II have been performed. The results are presented in Figs. 11, 12, 13 and 14. The samples I and II have been prepared from different parts of the composite. Therefore the distribution of the graphene oxide flakes could have been different. The differences in temperature response of the material sample to the heating process are visible in graphs also for samples without graphene oxide. The differences are less than 1% and may result from the inaccuracy of measurements. Figures 11 and 12 present the temperature distribution of rGO/HNBR composites in all heating time of 1 h while Figs. 13 and 14 show the results for NBR and FKM-based samples during the second half of the heating process.

As can be seen an increase in temperature of rGO/rubber composite compared to the matrix material temperature occurs. Differences in temperature distribution of samples with different graphene oxide content indicate an impact of the filler fraction on composites thermal conductivity.

A maximum temperature differences are about 11%. The constant temperature difference during the second half of heating time is observed and the thermal regular regime is achieved^32^. The received preliminary experimental results showed that the thermal properties of rGO/rubber composites improve with increasing of the graphene oxide content in the composite.

### Measurements with the guarded hot plate method

As in the preliminary investigations the thermal conductivity measurements have been performed for the rGO/rubber composites included in Table 1. For each material sample several tests have been made: for samples H0, H1, F1, F2 and N2—three measurements; for samples N0 and N1—four measurements. The mean sample temperature during tests *T*_*a*_ = (*T*_*m*_ + *T*_*u*_)/2 was in the range 25–26 °C. Temperature measurements have been performed with the use of the system of thermocouples type K which was the element of the applied GHP apparatus. The uncertainty of the temperature measurement method was equal to 2% while the uncertainty of the GHP method for thermal conductivity evaluation was 3%. Expanded uncertainties of thermal conductivity and temperature measurements have been estimated according to^33^. They were about 6.5% and 4% for thermal conductivity and temperature respectively.

As an example, the detailed results of four tests performed for N1 material sample are included in Table 2.

**Table 2:** Detailed results of thermal conductivity investigations in the case of rGO/NBR composite with graphene oxide concentration of 1.5%.

Number of measurement	$T_{u - } T_{m}$[K]	$T_{m - } T_{L}$[K]	$R_{int}$ × 10^4^ [$\frac{{{\text{m}}^{2} {\text{K}}}}{{\text{W}}}]$	$R_{s}$ × 10^2^ [$\frac{{{\text{m}}^{2} {\text{K}}}}{{\text{W}}}]$	*δ* [mm]	*k* [$\frac{{\text{W}}}{{{\text{mK}}}}]$	*T*_*a*_ [^o^C]
**Sample N1**							
1	14.33	4.72	4.752	2.24	7.65	0.341	25.44
2	14.16	4.81	4.750	2.15	7.65	0.355	25.92
3	14.21	4.69	4.751	2.22	7.65	0.344	25.49
4	14.92	5.14	4.750	2.13	7.65	0.359	26.09
The mean						0.350	25.7
Standard uncertainty of the mean						0.004	0.160
Uncertainty of the method, %						3%	2%
Uncertainty of the method						0.0105	0.5
Total uncertainty						0.0113	0.525
Expanded uncertainty, coverage factor = 2						0.023	1.05
Expanded uncertainty, %						6.5%	4.1%

**Figure 15:**
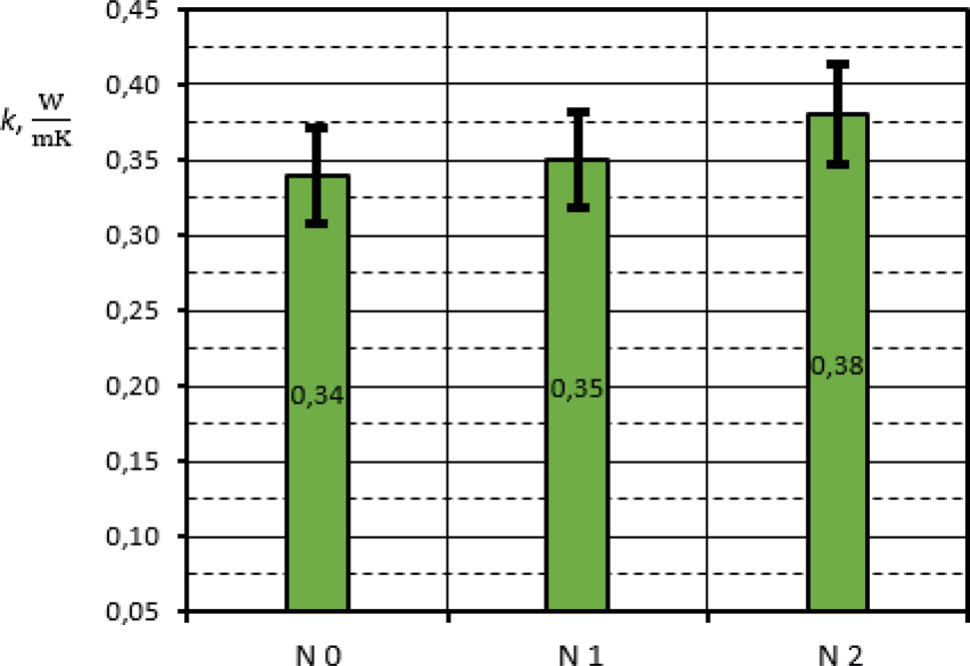
Thermal conductivity of rGO/NBR rubber composites in comparison of NBR matrix results.

**Figure 16:**
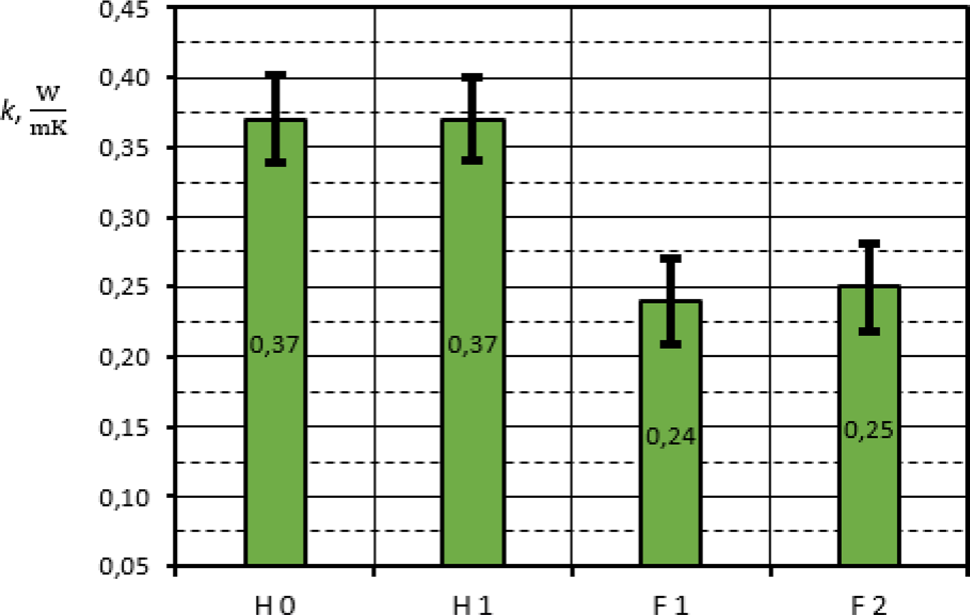
Thermal conductivity of rGO/HNBR and rGO/FKM rubber composites.

Measurement results for all composites tested with the use of GHP apparatus are summarized in Table 3. The graphical presentation of composites thermal conductivity results with the marked uncertainty bars is shown in Figs. 15 and 16.

**Table 3:** Thermal conductivity of rGO/rubber composites.

Sample	*k* $\left[ {\frac{{\text{W}}}{{{\text{mK}}}}} \right]$	Expanded uncertainty of *k* [%]	*T*_*a*_ [^o^C]	Expanded uncertainty of *T*_*a*_ [%]
N0	0.34	6.3	26.1	4.4
N1	0.35	6.5	25.7	4.1
N2	0.38	6.6	26.1	4.0
H0	0.37	6.2	26.0	3.9
H1	0.37	6.1	25.9	3.9
F1	0.24	6.2	25.2	4.0
F2	0.25	6.1	25.2	4.0

The investigation results received show that the addition of the reduced graphene oxide into different rubber matrix causes an increase of thermal conductivity. The visible changes in thermal conductivity are rather small and they are in the range of measurement uncertainty. However, the preliminary investigations in which the thermal regular regime characterized by the constant samples temperature differences has been achieved, confirm the influence of rGO addition on thermal conductivity. The maximum difference is observed in the case of rGO/NBR composites: N0 and N2, and it is about 11%. This result is comparable to the preliminary tests basing on the thermal regular regime theory. In turn the HNBR results are the same for H0 and H1 samples. However, the expected increase in thermal conductivity depending on graphene oxide content may be within the measurement error. It is similar in the case of FKM, where the differences in thermal conductivity are in the order of 4%.

### Thermal conductivity of rGO

On the basis of the results received for rGO/rubber composites thermal conductivity of the reduced graphene oxide applied has been estimated. Two classical models of effective thermal conductivity of two-component materials have been used. The first—ME model^34^ (Maxwell–Eucken) is given by the effective thermal conductivity equation in the form

5 $$k_{ef} = \frac{{k_{1} \varphi_{1} + k_{2} \varphi_{2} \frac{{3k_{1} }}{{2k_{1} + k_{2} }}}}{{\varphi_{1} + \varphi_{2} \frac{{3k_{1} }}{{2k_{1} + k_{2} }}}}.$$

The second EMT model^34^ (Effective Medium Theory) is given by

6 $$\varphi_{1} \frac{{k_{1} - k_{ef} }}{{k_{1} + 2k_{ef} }} = \varphi_{2} \frac{{k_{1} - k_{ef} }}{{k_{1} + 2k_{ef} }}.$$

In both mentioned above models *k*_*ef*_ represents thermal conductivity of the composite material, subscripts 1 and 2 represent continuous phase of the material and the dispersed phase, respectively.

Basing on the thermal conductivity of the N0 and N1 samples, using the formulas (5) and (6) and taking into account the suitable values of component volume concentrations *φ*_1_ and *φ*_2_, thermal conductivity of the rGO – *k*_2_ has been calculated. Application of the model ME gave the result *k*_2_ = 34.3 W/(mK). In turn from the model EMT—*k*_2_ = 21.1 W/(mK). The results received are in a good accordance with the literature results^35^ obtained for the graphene oxide with the use of non-equilibrium molecular dynamics method.

The use of classical structural models to determine thermal conductivity of rGO validated the thermal conductivity measurement method applied.

## Conclusions

In the present study, investigations of thermal properties of the reduced graphene oxide/rubber composites have been performed. As matrix materials NBR, HNBR and FKM rubbers have been used. The graphene oxide reduced with sodium hypophosphite has been applied as a filler material improving mechanical and thermal properties of the composite. The composites were investigated in terms of applications as a material of the roller bearing seals. Before thermal investigations the experimental research on some mechanical properties of the composites considered have been performed. The results of the investigations showed an improvement in elastic modulus and indentation hardness of the rGO/NBR and rGO/HNBR rubber composites with the increase of rGO concentration.

Thermal conductivity of the composites has been measured with the use of guarded hot plate technique. Detailed calculations of the measurement uncertainty of the method applied have been carried out. The preliminary qualitative investigations by comparatively method have been also performed. SEM analysis has been made and it showed that the tested composite samples contain agglomerates of rGO nanoparticles. On the basis of the studies conducted and the obtained results some conclusions can be formulated:

- Thermal conductivity of rGO/rubber composites increases with rGO concentration increasing, the maximum enhancement occurs in the case of NBR matrix material.
- Due to heterogeneity of the composites tested the measurement method used in investigations is very important. The size of the material sample is significant.
- The another important factor determining the correctness of results is taking into account the measurement uncertainty. In the present study the differences in some results received are in the range of the measurement uncertainty.
- Thermal conductivity results received with the use of guarded hot plate method are consistent with the results from comparatively method based on thermal regular regime. Thus, the results can be regarded as reliable.
- The improvement of the rGO/NBR thermal conductivity compared with the pure rubber is comparable with the literature results received by guarded hot plate method for the same graphene oxide content. The received compatibility concerns the results with taking into account measurement uncertainty.
- The use of classical structural models for two-component materials verifies the measurement method applied in the present work to determine thermal conductivity of rGO/rubber composites.

## List of symbols

*d*: Diameter [mm]
*E*: Elastic modulus [GPa]
*HIT*: Indentation hardness [MPa]
*k*: Thermal conductivity [W/(mK)]
*m*: Mass [kg]
*q*: Heat flux [W/m^2^]
*R*: Thermal resistance [m^2^K/W]
*T*: Temperature [°C, K]
*t*: Time [s]
*V*: Volume [m^3^]
*w*: Nanoparticle weight concentration [%]
*φ*: Volume concentration of the component [%]
*δ*: Thickness [m]
*ρ*: Density [kg/m^3^]

## Abbreviations

EDS: Energy-dispersive X-ray spectroscopy
EMT: Effective medium theory
FKM: Fluoroelastomer
GHP: Guarded heat plate
GO: Graphene oxide
HNBR: Hydrogenated acrylonitrile butadiene rubber
ME: Maxwell–Eucken model
NBR: Acrylonitrile butadiene rubber
phr: Parts per hundred rubber
rGO: Reduced graphene oxide
SEM: Scanning electron microscopy
XPS: X-ray photoelectron spectroscopy
